# Pyroptosis as a therapeutic target in preeclampsia: current research and future directions

**DOI:** 10.3389/fimmu.2025.1622550

**Published:** 2025-06-25

**Authors:** Yongchun Zhu, Yuting Xiang, Sathiskumar Swamiappan, Zhongjun Li, Xinsheng Peng

**Affiliations:** ^1^ School of Pharmacy, Guangdong Medical University, Dongguan, Guangdong Province, China; ^2^ Department of Obstetrics, the Tenth Affiliated Hospital, Southern Medical University, Dongguan, China; ^3^ Key Laboratory of Obstetrics and Gynecology for Major Diseases in Dongguan, Dongguan, China; ^4^ The First Clinical Medical College, Guangdong Medical University, Zhanjiang, China

**Keywords:** preeclampsia, Trophoblasts, pyroptosis, inflammation, NLRP3 inflammasome

## Abstract

Preeclampsia (PE) is a severe pregnancy-specific disorder characterized by new-onset hypertension and proteinuria after the 20th week of gestation, posing significant threats to maternal and fetal health. Globally, approximately 4 million women are diagnosed with PE annually, resulting in over 70,000 maternal deaths and 500,000 infant deaths. The exact pathogenesis of PE remains unclear and is associated with multiple factors, including obesity, diabetes, and chronic kidney disease. Pyroptosis, a newly discovered form of programmed cell death, is characterized by plasma membrane rupture and the release of numerous inflammatory mediators. Studies have shown that trophoblast pyroptosis is closely related to PE, potentially hindering trophoblast invasion, causing abnormal remodeling of uterine spiral arteries, and inducing systemic inflammatory responses. This review summarizes the latest research progress on the correlation between trophoblast pyroptosis and the pathogenesis of PE. It explores the regulatory roles of NLRP3 Inflammasome,oxidative stress, T helper type 1 (Th1)/T helper type 2 (Th2) cell imbalance, microRNAs and other factors in trophoblast pyroptosis, providing potential targets for the development of early diagnostic biomarkers and therapeutic strategies for PE.

## Introduction

**Figure 1:**
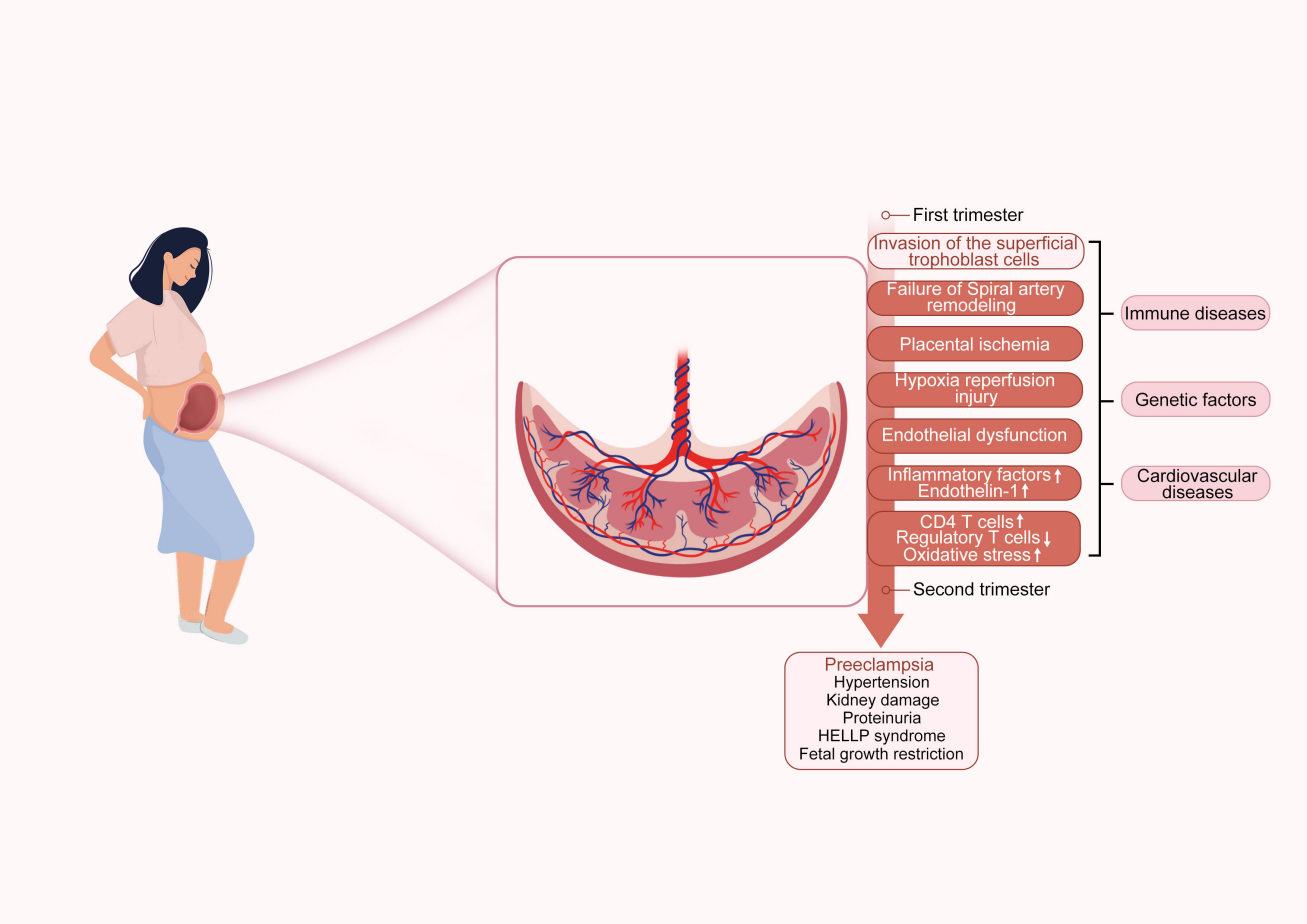
Preeclampsia pathogenesis diagram. The diagram illustrates the pathogenesis of PE, divided into early and late stages of pregnancy. The early stage includes insufficient trophoblast invasion, failure of spiral artery remodeling, placental ischemia, hypoxia reperfusion injury, and endothelial dysfunction. The late stage leads to complications such as PE, hypertension, kidney damage, proteinuria, HELLP syndrome, and fetal growth restriction. The diagram also indicates associations with PE-related immune diseases, genetic factors, and cardiovascular diseases.

Preeclampsia (PE) is a pregnancy-specific complication characterized primarily by hypertension and proteinuria, typically occurring after 20 weeks of gestation. This disease is an significant cause of maternal and fetal mortality (1). The diagnostic criteria for PE include the development of new-onset hypertension (defined as systolic blood pressure ≥140 mmHg and/or diastolic blood pressure ≥90 mmHg) after 20 weeks of gestation, as well as proteinuria or visceral organ dysfunction (2). This change in definition reflects a deeper understanding of the disease. Historically, PE was usually defined as the combination of hypertension and proteinuria, however it is now recognized that hypertension alone, coupled with significant visceral organ dysfunction, is also sufficient for diagnosis (3). Furthermore, the pathogenesis of PE is complex, involving multiple pathological processes (**Figure 1**). PE not only affects the health of pregnant women but also poses a serious threat to the fetus. Maternal complications may include hypertension, hepatic impairment, renal insufficiency, cerebral injury, and even death (3, 4). For the fetus, restricted maternal blood flow and nutrient supply may lead to risks such as intrauterine growth restriction, preterm birth, and fetal death (5). In terms of long-term health impacts, women who have experienced PE may face a higher risk of cardiovascular diseases and chronic hypertension later in life (6).

In addition, PE is one of the leading causes of maternal and perinatal mortality. It is estimated that worldwide, approximately 4 million women are diagnosed with PE annually, resulting in the deaths of over 70,000 women and 500,000 infants (7, 8). The occurrence of PE is closely related to a variety of risk factors (9). The mother’s health status is an important factor affecting the risk of PE. For example, research has shown that obesity may increase the risk of PE by influencing inflammatory responses, hormone levels, and vascular function (10, 11). In addition to women with pre-existing hypertension having a significantly increased risk of developing PE during pregnancy, women with pre-pregnancy diabetes, whether type 1 or type 2, may also have an increased risk of complications during pregnancy, which can trigger PE (12). Moreover, patients with chronic kidney disease also face a higher risk of PE, which may be related to the role of the kidneys in regulating blood pressure (13). In addition to maternal health status, family history is also associated with the risk of PE. If a mother or sister has experienced PE, the individual’s risk of developing the disease significantly increases, highlighting the importance of genetic factors in PE (13). Among pregnancy-related factors, the risk of multiple pregnancies is higher than that of singleton pregnancies, partly because placental growth and development are more complex (14, 15).

However, pyroptosis is a form of programmed cell death distinct from apoptosis and necrosis. The Gasdermin protein family mediates pyroptosis and depends on inflammatory caspases (16). The main characteristics of pyroptosis include plasma membrane bubbling, cytoplasmic swelling, rupture of the cell membrane, and the release of large amounts of inflammatory mediators, such as IL-1β and IL-18 (17). Studies have shown that in the placental tissues of patients with PE, the levels of active caspase-1 (CASP-1) and its substrates or cleavage products, Gasdermin D (GSDMD), Interleukin-1β (IL-1β) and Interleukin-18 (IL-18) are elevated and significantly higher than those in healthy controls. This indicates that trophoblast pyroptosis plays a vital role in the development of PE (18). Moreover, the mechanisms of pyroptosis in PE may involve multiple signaling pathways. For example, under hypoxia and endoplasmic reticulum stress conditions, the activation of the NLRP3 inflammasome can induce trophoblast pyroptosis (18). Additionally, the imbalance of T helper type 1 (Th1)/T helper type 2 (Th2) cell ratios may also promote trophoblast pyroptosis, thereby affecting the pathogenesis of PE. These findings provide a new perspective for understanding the immune-inflammatory mechanisms of pyroptosis in PE (19). In terms of maternal health, pyroptosis is associated with endothelial cell dysfunction. The release of inflammatory factors can lead to the activation and damage of endothelial cells, resulting in endothelial dysfunction. In PE, this manifests as increased vascular contraction, platelet aggregation, and thrombosis. These changes may lead to elevated maternal blood pressure and exacerbate the symptoms of PE (20). For the fetus, placental pyroptosis in PE affects maternal vascular function and the placenta’s blood supply. Inflammation and cell death in the placenta can lead to reduced placental function, affecting fetal development. The increased demand for maternal blood flow in the placenta, combined with reduced blood flow due to pyroptosis, may result in fetal hypoxia and growth restriction (21). Although there is a preliminary understanding of the mechanisms of pyroptosis in PE, further research is needed to elucidate its specific mechanisms and clinical significance.

## Preeclampsia pathophysiology

### Two-stage model of preeclampsia

The “two-stage” model is widely accepted as the pathophysiological mechanism of PE (22). The pathophysiological development of PE is generally understood as a two-stage process. In the first stage, also known as the preclinical stage, trophoblasts fail to adequately invade the uterine decidua, resulting in incomplete remodeling of the uterine spiral arteries, shallow placental implantation, and insufficient blood supply. This leads to placental ischemia and hypoxia (23). This condition stimulates the placenta to produce large amounts of factors, such as placental and vascular endothelial growth factors (24). In the second stage, the clinical phase, the ischemic and hypoxic condition of the placenta worsens, leading to placental tissue damage and cellular necrosis. This impairs the body’s antioxidant capacity, causing an imbalance between oxidation and antioxidation, and triggering an oxidative stress response (25). The byproducts of oxidative stress and placental factors enter the systemic circulation and affect the entire body, leading to clinical manifestations such as endothelial dysfunction, abnormal coagulation, imbalance of vasoactive substances, and lipid metabolism disorders (26).

### The role of immune cells and inflammatory factors in preeclampsia

Immune cells and mediators both play essential roles in the development of PE (27). During pregnancy, immune cells in the decidua are vital to the maternal-fetal interface. These immune cells include T cells, decidual natural killer cells, macrophages, and dendritic cells, which account for approximately 30% to 40% of the total decidual cells in early pregnancy (28). In normal pregnancy, the balance of T helper cells leans toward a Th2-type response, which is crucial for preventing immune attacks on the fetus. The cytokines produced by Th2-type immune responses, such as interleukin-4(IL-4)and interleukin-10(IL-10), enhance antibody-mediated immune responses, thereby protecting the fetus from maternal immune system attacks and maintaining immune tolerance in a dynamic equilibrium (29). However, in PE, this immune tolerance is disrupted, with increased immune system activation, leading to a significant reduction in regulatory T cells and a shift toward a Th1-dominant response, thereby reducing maternal immune tolerance to the embryo. This imbalance in immune tolerance is associated with the pathogenesis of PE, particularly in the maternal immune response to the placenta (30).

## Molecular mechanisms of pyroptosis

**Figure 2:**
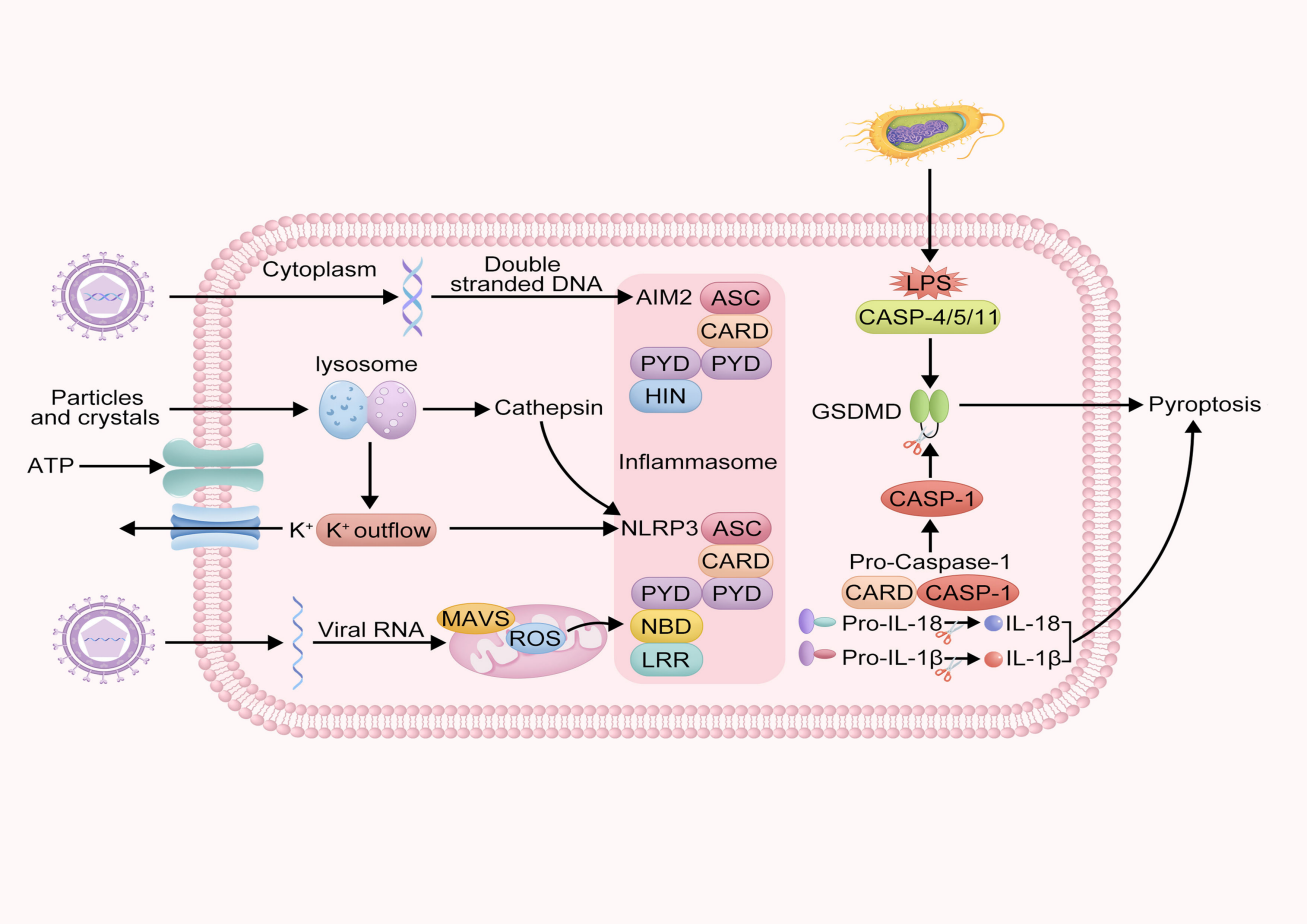
Schematic of intracellular inflammasome activation and pyroptosis mechanism. This diagram describes the molecular mechanisms of intracellular inflammatory responses and pyroptosis. It illustrates how pathogen-associated molecules such as double-stranded DNA and LPS activate AIM2 and NLRP3 inflammasomes, activating CASP-1, leading to the cleavage of GSDMD protein and triggering pyroptosis. Additionally, the diagram shows the lysosomal pathway and the process of MAVs and viral RNA activating the NLRP3 inflammasome.

Pyroptosis is a form of programmed cell death accompanied by an inflammatory response. Depending on the type of caspase involved, pyroptosis can be classified into canonical and non-canonical pathways (**Figure 2**). The canonical pathway of pyroptosis mainly relies on the activation of CASP-1. Under stimulation by bacterial, viral, or other signals, inflammasomes form and activate CASP-1. Activated CASP-1 cleaves GSDMD to form the N-terminal fragment of GSDMD, creating pores in the cell membrane. It also cleaves the precursors of IL-1β and IL-18 to form active IL-1β and IL-18, both of which are released as inflammatory mediators into the extracellular space, thereby amplifying the inflammatory response (31). The inflammasome is a multiprotein complex within the cell, primarily composed of pattern recognition receptors, such as NOD-like receptors and absent in melanoma 2, as well as apoptosis-associated speck-like protein (ASC) and pro-CASP-1 (32).

However, the non-canonical pathway of pyroptosis primarily relies on activating caspase-4/5/11 (CASP-4/5/11). Under stimulation by bacterial signals, human CASP-4 and CASP-5, and murine CASP-11 can directly recognize lipopolysaccharide (LPS) within the cell. The acylated lipid A component of LPS binds to the Caspase Recruitment Domain (CARD) domain of pro-CASP-4/5/11, forming a non-canonical inflammasome. CASP-4, -5, and -11 are activated and cleave GSDMD to form the N-terminal fragment of GSDMD, which creates pores in the cell membrane, leading to pyroptosis (33). Additionally, studies have shown that the membrane hemichannel protein pannexin-1 can open its channel to release intracellular Adenosine triphosphate (ATP) into the extracellular space or allow extracellular ATP to enter the cell, thereby participating in the non-canonical pathway (34, 35).

## Regulation of pyroptosis in preeclampsia

### Regulation of pyroptosis by the nlrp3 inflammasome in preeclampsia

The NLR family protein 3 (NLRP3) inflammasome is a multiprotein complex within the cell, composed of NLRP3, the adaptor protein ASC, and the protease CASP-1 (36). The activation of the NLRP3 inflammasome is a two-step process. First, the priming phase involves the activation of the NF-κB signaling pathway by Toll-like receptor ligands, cytokines, and other factors, which upregulates the expression of NLRP3 and IL-1β, laying the foundation for subsequent activation (37). Subsequently, upon stimulation by various activating signals, NLRP3 undergoes oligomerization, recruiting ASC and pro-CASP-1 to form an active inflammasome complex, activating CASP-1 (38). The activation of the NLRP3 inflammasome depends on multiple stimulating signals, including potassium ion efflux, Nuclear Factor kappa-light-chain-enhancer of activated B cells (NF-κB), autophagy, and mitophagy (39). Additionally, the activation of NLRP3 relies on its phase separation properties. Studies have shown that the phase separation of NLRP3 depends on palmitoylation mediated by Zinc finger, DHHC-type containing 7 and exhibits liquid-liquid phase separation characteristics. This phase separation process involves the intrinsically disordered region in the Fish-specific NACHT-associated domain of NLRP3, where conserved hydrophobic residues mediate multivalent weak interactions, promoting the phase separation and activation of NLRP3 (40).

Activating the NLRP3 inflammasome directly leads to pyroptosis, a form of inflammatory cell death. Activated NLRP3 inflammasomes can activate the protease CASP-1, which in turn induces gasdermin-dependent pyroptosis and promotes the release of IL-1β and IL-18 (41). The release of these cytokines is essential for innate immune defense and homeostasis maintenance, but their overactivation is closely related to the development of chronic inflammatory diseases (42). The hallmarks of pyroptosis, including the release of cellular contents and the secretion of inflammatory factors, are consistent with the cellular responses following inflammasome activation (43). Moreover, various cell death effectors can also regulate the activation of the NLRP3 inflammasome, indicating a close relationship between cell death and inflammasome activation (44).

In PE, the overactivation of the NLRP3 inflammasome is one of the key pathological mechanisms. Studies have shown that the activity of NLRP3, CASP-1, and GSDMD is significantly increased in the placental tissues of PE patients, which is closely related to the overactivation of the inflammasome (45). Multiple factors, including hypoxia, endoplasmic reticulum stress, and the unfolded protein response, may contribute to this overactivation (46). Hypoxia is one of the characteristic pathological features of PE and activates the NLRP3 inflammasome through various pathways (47). Under hypoxic conditions, the expression of NLRP3, CASP-1, and GSDMD in placental trophoblasts is significantly increased, along with elevated levels of the inflammatory factors IL-1β and IL-18 (48). Additionally, hypoxia can promote the activation of the NLRP3 inflammasome by activating Thioredoxin-interacting protein (49).

### Regulation of pyroptosis by oxidative stress in preeclampsia

Oxidative stress is defined as the imbalance between the generation and accumulation of reactive oxygen species (ROS) in cells and tissues and the capacity of antioxidant mechanisms to neutralize these reactive species (50). In the pathogenesis of PE, oxidative stress plays a pivotal role, significantly driving disease progression through multiple mechanisms (51). First, PE is frequently associated with elevated oxidative stress and the accumulation of ROS (52). Excessive ROS not only disrupt normal mitochondrial function (53), leading to abnormalities in the electron transport chain and the accumulation of superoxide anion and hydrogen peroxide (54), but also weaken the cellular antioxidant defense system, particularly by downregulating Nuclear factor erythroid 2-related factor 2 (Nrf2) expression, thereby reducing the cell’s ability to clear ROS (55). ROS can also attack cell membranes, inducing lipid peroxidation and generating toxic products such as malondialdehyde and 4-hydroxynonenal, further damaging cell structure and function (56).

Oxidative stress directly activates the NLRP3 inflammasome through multiple pathways. ROS oxidize the thiol groups of NLRP3, promoting its binding to NIMA-related kinase 7 and thereby activating the inflammatory response (57). Additionally, oxidative stress induces the release of mitochondrial DNA, typically through the opening of the mitochondrial permeability transition pore (58). These events collectively activate CASP-1, which cleaves GSDMD to form membrane pores, triggering pyroptosis and the release of IL-1β and IL-18, thereby amplifying the inflammatory response (59–61). Clinical studies have confirmed that the expression of NLRP3, CASP-1, and GSDMD is significantly elevated in the placental tissues of PE patients, indicating that oxidative stress-induced inflammation and pyroptosis play essential roles in the pathogenesis of PE (62). Moreover, antioxidants such as N-acetylcysteine can reduce pyroptosis, further confirming the role of oxidative stress in PE (63).

Several key regulatory pathways are closely related to oxidative stress in the pathogenesis of PE. The Long Intergenic Non-Protein Coding RNA 240/MicroRNA-155/Nrf2 axis is an important regulatory network, in which MicroRNA-155 enhances oxidative stress by inhibiting Nrf2 expression. Simultaneously, Long Intergenic Non-Protein Coding RNA 240 antagonizes the effect of MicroRNA-155, thereby regulating Nrf2 expression and activity (64). Additionally, PTEN-induced putative kinase 1/Parkin-mediated mitophagy is crucial for maintaining mitochondrial health and function; defects in mitophagy can lead to ROS accumulation and exacerbated oxidative stress (65). The synthesis of ceramide is also regulated by ROS, which promotes ceramide synthesis by activating sphingomyelinase, and ceramide can directly activate the NLRP3 inflammasome, further driving the inflammatory response and pyroptosis (66).

### Regulation of pyroptosis by Th1/Th2 cell imbalance in preeclampsia

The pathogenesis of PE is complex, involving multiple pathological processes, among which immune imbalance and abnormal pyroptosis of placental trophoblasts are key features (67). Th1 cells secrete cytokines such as interferon-gamma (IFN-γ), which can activate inflammatory signaling pathways, including the NLRP3 inflammasome pathway. Activation of the NLRP3 inflammasome leads to the activation of CASP-1, which cleaves GSDMD into its active form, triggering pyroptosis and exacerbating the inflammatory response. In contrast, Th2 cells secrete anti-inflammatory cytokines such as IL-10 and IL-4, which can inhibit inflammatory responses. In PE patients, the levels of these anti-inflammatory cytokines are significantly reduced, failing to effectively suppress pyroptosis-inducing factors related to inflammation (68–70). Studies have confirmed that this immune imbalance may further exacerbate the pathophysiological processes of PE, leading to increased inflammation and pyroptosis. For example, in PE patients, the immune imbalance is characterized by an elevated Th1/Th2 ratio (Th1 predominance), with significant increases in T-bet expression in peripheral blood Cluster of Differentiation 4-positive T cells (71), increased IFN-γ expression in placental tissues compared to normal pregnancies (72), and reduced IL-4 and IL-10 cells in the decidua (73). Th1-type factors may play a significant role in PE. For instance, in studies of other diseases, IFN-γ has been shown to upregulate NLRP3 inflammasome genes by activating Signal Transducer and Activator of Transcription 1, thereby promoting CASP-1 activation (74), while Tumor Necrosis Factor-alpha synergizes with hypoxia-induced Hypoxia-Inducible Factor 1-Alpha to enhance GSDMD transcription (75). However, Th2-type factors such as IL-4 can inhibit the assembly of the NLRP3 inflammasome, reducing its mediated inflammatory response (76).

### Regulation of pyroptosis by microRNAs and other factors in preeclampsia

MicroRNAs are ubiquitously present in mammalian cells and constitute a class of non-coding single-stranded RNAs encoded by endogenous genes, with lengths ranging from 18 to 24 nucleotides (77). MicroRNAs play important roles in pyroptosis and PE-related biological pathways. For example, melatonin may inhibit HtrA serine peptidase 1 transcription through the MicroRNA-520c-3p/SET domain containing (lysine methyltransferase) 7 axis, thereby promoting the invasion and migration of trophoblasts in PE and reducing trophoblast pyroptosis (78). Similarly, MicroRNA-223-3p inhibits the activation of the NLRP3 inflammasome, the secretion of downstream inflammatory factors, and pyroptosis in LPS-induced HTR8/SVneo cells, indicating that miR-223-3p can function as an anti-inflammatory factor in PE (79). MicroRNA-124-3p is upregulated in PE and targets placental growth factor to suppress the proliferation, migration, and invasion of trophoblast HTR-8/SVneo cells while promoting trophoblast pyroptosis (80).

Beyond the aforementioned mechanisms, other placental factors may also participate in the regulation of pyroptosis. For example, a study using a PE mouse model and treating human first-trimester villi with Interleukin-11(IL-11) demonstrated that IL-11 activates placental inflammasomes, resulting in villous pyroptosis in human placentas and PE in the mouse model (81). Metformin suppresses Toll-like receptor 4/NF-κB/6-phosphofructo-2-kinase/fructose-2,6-bisphosphatase 3 signaling pathways, correcting glucose metabolic reprogramming in trophoblasts and NLRP3 inflammasome-induced pyroptosis, demonstrating potential therapeutic value (82). Furthermore, a study employing hypoxia/reoxygenation models to stimulate human and rat trophoblasts revealed that under H/R conditions, chemerin expression is upregulated via Homeobox A9. Chemerin subsequently activates the Chemerin Chemokine-Like Receptor 1/AMP-activated protein kinase/Thioredoxin Interacting Protein/NLRP3 inflammasome pathway, thereby promoting trophoblast pyroptosis and inflammation and exacerbating PE (83). Urotensin II levels in the placentas of PE patients are positively correlated with pyroptosis markers. This suggests that Urotensin II may promote pyroptosis in PE, thereby amplifying inflammation and impairing normal placental development and function, thus exacerbating PE (84).

## Potential applications of pyroptosis in the diagnosis and treatment of preeclampsia

### Biomarker detection

Pyroptosis plays a crucial role in the pathogenesis of PE, and its related molecules can serve as potential biomarkers for early diagnosis, disease monitoring, and prognosis assessment. For instance, studies have shown that the NLRP3 inflammasome and ASC expression are significantly elevated in PE patients’ placental tissues. Activation of the NLRP3 inflammasome promotes the release of downstream pro-inflammatory factors such as IL-1β and IL-18, which may serve as potential diagnostic biomarkers and positively correlate with disease severity. Detection methods include immunoblotting, immunohistochemistry, and quantitative real-time PCR to quantify or qualitatively analyze NLRP3 and ASC in serum or placental tissues, providing a basis for disease classification and prognosis assessment (85). Compared with other biomarkers, IL-1β and IL-18 have the advantage of mature detection methods (e.g., routine Enzyme-Linked Immunosorbent Assay) and high feasibility for clinical translation (86).

Beyond these specific markers, some non-specific molecules associated with pyroptosis may also serve as auxiliary diagnostic tools for PE. For example, lactate dehydrogenase, a general marker of cell damage, is often elevated in PE patients due to placental cell pyroptosis. However, it lacks specificity and should be used in combination with other indicators for comprehensive judgment (87). Studies have suggested that incorporating lactate dehydrogenase and uric acid measurements into routine clinical practice may aid in early detection and intervention, ultimately improving outcomes in pregnancies complicated by PE (88). Additionally, the high-mobility group box 1, a damage-associated molecular pattern released by pyroptotic cells, can further amplify inflammatory responses and promote vascular endothelial damage in PE. Its levels may serve as a novel indicator for predicting disease severity (89). Future research could further develop biological markers such as mitochondrial DNA and GSDMD fragments to more accurately monitor the state of pyroptosis (90). However, there is currently a lack of biomarkers that specifically reflect pyroptosis in PE, increasing the difficulty of clinical diagnosis and posing challenges for research. Therefore, identifying and validating specific pyroptosis biomarkers is crucial, as they can provide a basis for early diagnosis and disease monitoring in PE. Integrating large-scale clinical sample analysis with basic research holds promise for discovering new biomarkers that accurately reflect the state of pyroptosis in PE (91).

### Therapeutic approaches targeting pyroptosis pathways

The mechanisms underlying pyroptosis in PE have gradually been elucidated, and therapeutic strategies targeting its regulatory pathways have demonstrated significant potential. For example,research on NLRP3 inflammasome inhibitors for treating PE is gradually revealing their potential. These inhibitors reduce the activation of the NLRP3 inflammasome, effectively decreasing the occurrence of pyroptosis, which is crucial for controlling the inflammatory response in PE. NLRP3 activation leads to the production of pro-inflammatory cytokines such as IL-1β, exacerbating the condition (92). Notably, 1,25-dihydroxyvitamin D3 protects the placenta from inflammation by inhibiting NLRP3-mediated IL-1β production and activating the Nrf2 signaling pathway (93). Additionally, MCC950 sodium, an inhibitor of the NLRP3 inflammasome, can directly suppress the inflammatory response (94). CASP-1 inhibitors, another potential therapeutic strategy, reduce pyroptosis by preventing the cleavage of GSDMD. CASP-1 plays a central role in the processing and release of pro-inflammatory cytokines such as IL-1β, and its increased activity in the placentas of PE patients highlights the potential therapeutic effects of CASP-1 inhibitors (95). GSDMD is a promising target in precision medicine, with broad applications in treating inflammation-related diseases and cancer. Its potential application in PE treatment also provides an important theoretical basis for future research (96). Therapeutic strategies targeting oxidative stress show significant potential in the management of PE. For example, resveratrol reduces oxidative stress by scavenging ROS (97). Exploring combination therapies of antioxidants and anti-inflammatory drugs, as well as personalized treatment plans, may help more effectively manage and treat PE (98, 99). Therapeutic strategies targeting Th1/Th2 cell imbalance have also shown great potential in the treatment of PE. For example,magnesium sulfate may inhibit pyroptosis by affecting the production of Th2 cytokines, such as blocking Ca²^+^ influx, thereby indirectly influencing Th2 cell activity and reducing Th2 cytokine levels (100, 101). Low molecular weight heparin can improve immune imbalance and reduce inflammatory responses by modulating Th1/Th2 cytokine levels. These findings provide important evidence for the application of low molecular weight heparin in immune regulation and the treatment of inflammation-related diseases (102). Future research directions include the development of nanobodies targeting Interleukin-6, which indeed demonstrates the potential of nanobodies in targeting cytokines. This provides a theoretical basis for developing nanobodies targeting other cytokines, such as Th1-polarizing IFN-γ. Specifically, the development of anti-IFN-γ nanobodies could leverage the synthetic phage display library technology mentioned in the study for rapid screening and generation of high-affinity nanobodies (103). Additionally, metformin, which dually regulates 5’-AMP-activated protein kinase/Hypoxia-Inducible Factor 1-Alpha, enhances autophagy and angiogenesis and reduces inflammatory responses, has shown therapeutic potential in wound healing in diabetic rats. This finding suggests that metformin may also have therapeutic potential in PE (104). Organoid models used to study various diseases, such as inflammatory bowel disease, colorectal cancer, and liver disease, may provide new insights for the treatment of PE (105). In summary, therapeutic strategies targeting pyroptosis and its regulatory pathways hold significant value in the management of PE. Future research needs to further explore their molecular mechanisms and clinical applications.

## Summary and outlook

This review summarizes the regulatory role of pyroptosis in PE and its potential diagnostic and therapeutic applications. PE is a severe pregnancy-specific disorder with a complex pathogenesis involving multiple pathophysiological processes, including abnormal placental development, immune imbalance, oxidative stress, and inflammatory responses. In recent years, pyroptosis, a novel form of programmed cell death, has been found to be closely related to the occurrence and development of PE. Studies have shown that pyroptosis of trophoblasts can hinder their invasive capacity, lead to abnormal remodeling of uterine spiral arteries, and induce local-to-systemic inflammatory responses, thereby promoting the development of PE.

The regulatory mechanisms of pyroptosis involve multiple signaling pathways and molecules. The activation of the NLRP3 inflammasome is a key step in pyroptosis, which activates CASP-1 to cleave GSDMD, forming pores in the cell membrane and releasing inflammatory factors such as IL-1β and IL-18, thereby amplifying the inflammatory response. Oxidative stress is significantly elevated in PE and can activate the NLRP3 inflammasome through various pathways, promoting pyroptosis. Additionally, Th1/Th2 cell imbalance, microRNAs, and other factors, such as IL-11 and Urotensin II, also play important roles in the regulation of pyroptosis. In terms of diagnosis and treatment, pyroptosis-related molecules such as NLRP3, ASC, IL-1β, and IL-18 can serve as potential biomarkers for early disease diagnosis and monitoring disease progression. NLRP3 inflammasome inhibitors, CASP-1 inhibitors, and antioxidants have shown promising therapeutic effects in animal models, providing new insights for the clinical treatment of PE.

Although the mechanisms underlying pyroptosis in PE have been preliminarily elucidated, many unresolved questions remain. Future research directions should focus on further investigating the functions and regulatory mechanisms of pyroptosis-related molecules to clarify their specific roles in PE. For example, techniques such as gene editing and proteomics can be employed to explore the activation mechanisms of the NLRP3 inflammasome and its interactions with other signaling pathways. There is a need to identify and validate specific biomarkers that reflect the state of pyroptosis in PE to enhance the accuracy of early disease diagnosis and disease monitoring. Combining large-scale clinical sample analysis with basic research may lead to the discovery of new biomarker combinations that provide stronger support for clinical applications. Research should also explore the combined use of antioxidants, anti-inflammatory drugs, and existing PE treatment regimens to investigate their potential value in improving disease outcomes. For instance, developing combination therapies targeting the NLRP3 inflammasome, oxidative stress, and immune imbalance may offer a more comprehensive solution for PE treatment. Expanding the research scope to include other pregnancy-related disorders, such as placental abruption and fetal growth restriction, can help explore the mechanisms and therapeutic potential of pyroptosis in different pathological states. Finally, strengthening the translation from basic research to clinical application is essential to promote clinical trials of pyroptosis-related diagnostic biomarkers and therapeutic drugs and accelerate their integration into clinical practice.

In conclusion, research on pyroptosis in PE is still in its developmental stage, and more studies are needed to deepen our understanding of its mechanisms and translate this knowledge into effective diagnostic and therapeutic tools in clinical practice. With continued research, it is hoped that new breakthroughs will be achieved in the prevention, diagnosis, and treatment of PE, ultimately improving maternal and fetal health outcomes.
